# Cognition: Memory perseverance through binding

**DOI:** 10.1038/s44271-023-00009-w

**Published:** 2023-08-15

**Authors:** W. Fred Garvey, Claudia C. von Bastian

**Affiliations:** Department of Psychology, University of Sheffield, Sheffield, UK

**Keywords:** Cognitive neuroscience, Human behaviour, Learning and memory

## Abstract

Are working memory representations that are no longer relevant actively deleted? A new study in *Attention, Perception, & Psychophysics* suggests that this isn’t the case: irrelevant memoranda linger on, especially when people create an imaginary combination of items they encounter.

**Figure:**
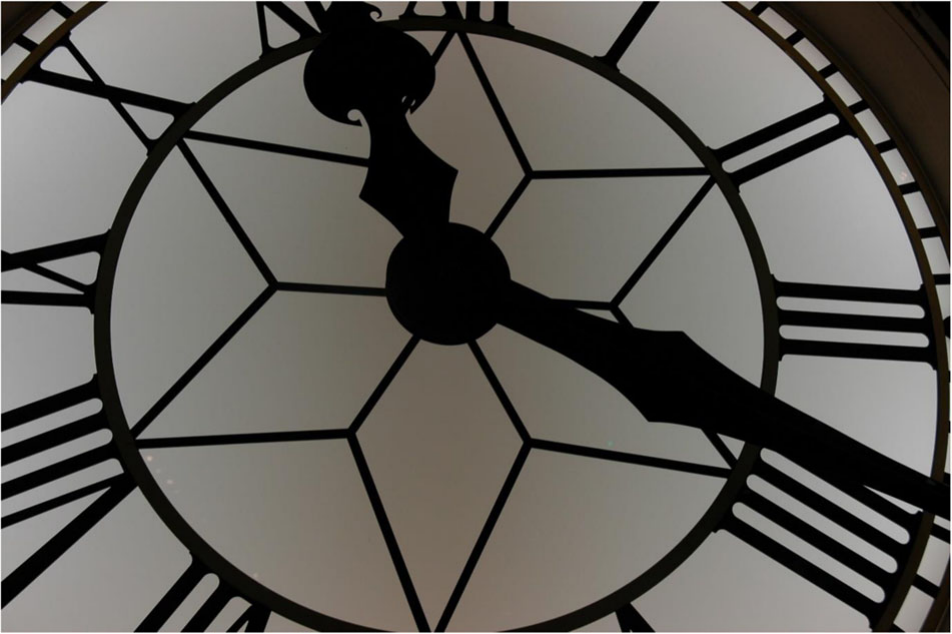
Jeanne Rouillard on Unsplash

Working memory (WM) is a system for goal-oriented processing of memory representations but has a limited capacity. Removing irrelevant memoranda may make valuable space in WM. What happens to your memories when they are not needed anymore? Are they gone forever, or do they linger and, if so, why?

Rhilinger and colleagues^1^ at the University of Notre Dame addressed this question by performing experiments using stimuli which interfere with each other if simultaneously stored in WM. They compared the amount of interference during recall as an indication of the items stored in WM and, thus, whether an item was deleted. The stimuli were two sets of slanted lines with orientations that had to be memorized and later reproduced. Either each stimulus was tested once, or one stimulus was tested twice. However, before each test, a cue signified which stimulus would be tested in that instance. Hence, for the second test, it was clear which stimulus was no longer needed to be retained in memory. If participants actively delete these no longer relevant representations, there should be less interference in the second test compared to the first when each stimulus was tested once. Surprisingly, Rhilinger et al. found the opposite: significantly more interference was observed during the recall of the second stimulus.

To better understand this finding, Rhilinger and colleagues conducted a second experiment in which they simply added the instruction to imagine the two orientations together as an angle or hands of a clock. Based on anecdotal evidence of participants’ strategies in their first experiment, the authors proposed that multiple stimuli mentally bound into a single object allow irrelevant stimuli to hang on to the relevant ones and stave off their deletion. Their results supported their hypothesis. When the degree differences between the orientations were considered, more interference was observed in the second test of this experiment thanin the second test of the first experiment – suggesting that mentally binding stimuli increased interference, possibly by preventing deletion.

Through clever analysis and use of anecdotal evidence from their first experiment, Rhilinger and colleagues made lemonade from lemons. They did not find evidence for active deletion but a mechanism for WM preservation – WM binding. Indeed, WM binding increases the information within a single WM representation by chunking individual memoranda together; Rhilinger and colleagues have shown that these memoranda are stronger together than alone.
